# Foliar polystyrene nanoplastics modulate cadmium uptake and root-to-shoot translocation in hydroponic *Spinacia oleracea L.*: Time- and concentration-dependent responses

**DOI:** 10.1371/journal.pone.0358424

**Published:** 2026-10-08

**Authors:** Mohamad Kazem Dehghani, Reza Ghasemi-Fasaei, Sajjad Abbasi, Amin Mojiri

**Affiliations:** 1 Department of Soil Science, School of Agriculture, Shiraz University, Shiraz, Iran; 2 Department of Earth Sciences, College of Science, Shiraz University, Shiraz, Iran; 3 School of Sustainable Engineering and the Built Environment, Arizona State University, Tempe, Arizona, United States of America; IIT Roorkee: Indian Institute of Technology Roorkee, INDIA

## Abstract

The accumulation of plastics and heavy metals (HMs) in the environment has become a significant global concern due to their persistence, ubiquity, and potential ecological impacts. Atmospheric micro- and nanoplastics (MP, NP) are capable of undergoing long-range transport and can be deposited onto plant shoots through precipitation, thereby exposing aerial plant tissues to these particulate contaminants. In this study, the effects of varying concentrations of polystyrene nanoplastics (PSNPs) (0, 10, 50, 100, 200, and 400 mg L^-1^), applied via foliar spraying, and cadmium (Cd) (0 and 5 mg L^-1^) were evaluated on the growth parameters and chemical composition of spinach (*Spinacia oleracea L.*) grown under hydroponic conditions. The experiment was carried out over three exposure durations—2, 4, and 6 weeks—designated as t_1_, t_2_, and t_3_, respectively. Fifteen-day-old seedlings were transplanted into hydroponic containers and sampled at the end of each exposure period. The results demonstrated that PSNPs had a significant impact on plant growth and biomass accumulation. At t_3_, exposure to 200 mg L^-1^ of PSNPs resulted in an 83.33% and 87.34% increase in the dry weight of shoots and roots, respectively, compared to the control. However, this positive trend reversed at higher concentrations; at 400 mg L^-1^, shoot and root dry weights decreased by 49.87% and 27.87%, respectively, relative to the 200 mg L^-1^ treatment. Cd concentration in the shoot at t_3_ increased by 72.28% at 50 mg L^-1^ PSNPs compared to the control but declined by 31.32% at 400 mg L^-1^. The Cd translocation factor (TF) from root to shoot rose sharply with increasing PSNPs concentrations, reaching a peak increase of 194% at 400 mg L^-1^ at t_3_. Additionally, TF values exhibited a positive correlation with plant age, suggesting enhanced TF in older plants. These results highlight the potential risk of PSNPs in Cd-contaminated environments, where they may modify Cd uptake, distribution and root-to-shoot translocation in spinach plants. By facilitating Cd transfer to shoots, particularly under prolonged exposure, PSNPs could increase the likelihood of Cd accumulation in edible tissues. This interaction raises concerns regarding the safety of leafy vegetables grown in agricultural systems co-contaminated with MPs/NPs and HMs.

## 1. Introduction

Plastic pollution is one of the most critical environmental challenges of our time, with millions of tons of waste entering ecosystems annually. In 2022, global plastic production reached 380 million tons, reflecting a 5.5% increase compared to 2021 [1]. More than 60% of the plastic produced ends up in landfills or natural environments, exacerbating global plastic pollution [2]. Among various types of plastic waste, microplastics (MPs) and nanoplastics (NPs) are of particular concern due to their significant threats to both environmental and human health [3]. Large plastic debris released into the environment gradually degrades into smaller fragments through physical, chemical, and biological processes [4–6]. The final size of these plastic particles depends on the polymer type, environmental conditions, and other contributing factors. Based on their diameter, plastic particles are generally classified as MPs (<5 mm) [7,8] and NPs. NPs are defined as plastic particles with at least one external dimension in the range of approximately 1–1000 nm (≤1 µm); however, in some classification frameworks, the term “nano” is restricted to particles smaller than 100 nm, while particles in the 100–1000 nm range are considered sub-micron plastics [9].

Agricultural crops are persistently exposed to such particles throughout their growth cycle, which can enter plant tissues through both roots and shoots [10]. While the plant cell wall acts as a barrier against MPs due to their relatively larger size [11], NPs, owing to their much smaller dimensions, can bypass this barrier and directly infiltrate plant cells [12]. Experimental evidence confirms the uptake and accumulation of NPs in various plant species, including *Lactuca sativa* [13], *Triticum aestivum* [14], *Arabidopsis thaliana* [15], and *Allium cepa* [16]. The toxicity of MPs and NPs in plants is influenced by several factors, including polymer type, particle size, shape, plant species, and soil conditions [17–20]. Studies indicate that these plastic particles can adversely affect seed germination, photosynthesis, and plant growth, as well as induce oxidative stress in plant cells [21]. Plants exhibit diverse physiological and biochemical responses to the stress induced by MP and NP exposure, highlighting the need for further research into their long-term ecological implications.

Environmental pollution induced by heavy metals (HMs), including arsenic, chromium, copper, lead, and cadmium (Cd), adversely impacts plant growth and development [22]. The extent of these harmful effects depends on several factors, including plant species, the specific HM involved, soil microbial communities, and the physicochemical properties of the soil, all of which influence metal accumulation in plant tissues [23–26]. Elevated HM concentrations can lead to stunted growth, leaf wilting, and discoloration, often manifesting as a blue-purple hue [21]. Additionally, HMs primarily affect plant roots—the first site of exposure—by inhibiting root elongation, cell division, and overall root system development [27,28].

Cd contamination in soil represents a significant global challenge in agriculture and poses a serious public health risk due to its harmful impacts on human health and environmental quality [29]. Cd uptake by plant roots disrupts essential physiological processes, particularly water and nutrient absorption [30], leading to impaired root development and reduced overall plant performance. Additionally, by impairing chlorophyll synthesis, Cd interferes with photosynthesis and consequently hampers plant growth and developmental processes [31]. The combined impact of Cd-induced root damage, photosynthetic inhibition, and nutrient imbalance results in a substantial decline in plant biomass. These adverse effects not only reduce yield but also contribute to soil degradation, further exacerbating agricultural and ecological challenges.

MPs and NPs, as pervasive forms of plastic pollution, represent substantial challenges to environmental integrity and ecosystem health. The co-occurrence of plastic particles and HMs in the environment can lead to compounded effects on plant development and physiological function [32,33]. MPs and NPs may enhance the mobility and bioaccessibility of HMs by functioning as transport vectors, which can ultimately lead to increased accumulation of these metals in plant tissues [2,34]. In addition, they can influence HM adsorption–desorption dynamics, with their sorption behavior largely dependent on the physicochemical properties of both the plastic particles and the metals involved [35]. These particles are mainly absorbed through root fibers, resulting in the internal accumulation of Cd in various plant tissues [36]. NPs have been shown to modulate plant responses to Cd stress. Some research findings suggest that polystyrene nanoplastics (PSNPs) may mitigate Cd-induced toxicity in crops like wheat by limiting Cd translocation to the leaves [21]. Conversely, the interaction between plastic particles and Cd can intensify oxidative stress in plants, as indicated by elevated levels of reactive oxygen species (ROS) and malondialdehyde (MDA), a biomarker of lipid peroxidation [37]. These biochemical alterations compromise photosynthetic efficiency and adversely affect overall plant health. NPs interacting with HMs represent a dual threat, endangering both plant health and broader ecological and food safety systems. The bioaccumulation of HMs in consumable plant tissues increases the likelihood of their transfer through the food chain, with potential adverse effects on human health [38,39]. Additionally, the combined toxic effects of plastic particles and HMs exacerbate environmental hazards and present substantial challenges for remediation strategies [21].

This study addressed one of the less explored aspects of PSNPs, namely the effects of airborne NPs on plant growth and HM uptake. In this study, by using a hydroponic culture system, the interfering and complex effects of the soil environment were eliminated to enable a more precise evaluation of the consequences of foliar application of NPs. While most previous studies have mainly focused on MPs in the soil-based systems, and the entry of these particles through the root zone, the effects of foliar application of NPs under conditions of simultaneous plant exposure to HMs such as Cd have received less attention.

In the present study, spinach (*Spinacia oleracea* L., Hachure variety) was selected as the plant due to its fast growth rate, sensitivity to environmental stressors, and high capacity for accumulating HMs and NPs. Given its nutritional importance, evaluating the impact of environmental pollutants on this crop is of particular relevance. Accordingly, this study was designed to achieve the following objectives:

- To evaluate the impact of various concentrations of NPs on the spinach growth;
- To examine the effect of different Cd levels on spinach growth;
- To investigate the interactive effects of NPs and Cd on plant performance;
- To assess how the duration of exposure to these pollutants influences the severity of their impacts on the plant.

## 2. Materials and methods

### 2.1. Physicochemical analysis of PSNPs

PSNPs were obtained from the Institute for Color Science and Technology for further analysis. Their morphological features were assessed via scanning electron microscopy (SEM) using a TESCAN-Vega3 device, and representative micrographs were captured (Fig 1). The hydrodynamic diameter (Z-average) and polydispersity index (PDI) were determined by dynamic light scattering (DLS) using an SZ-100 analyzer, and the zeta potential of the PSNPs was also measured using the same instrument. The chemical structure and identity of the PSNPs were verified by fourier transform infrared spectroscopy (FTIR), performed with a Bruker TENSOR II instrument (Fig 2).

**Fig 1:**
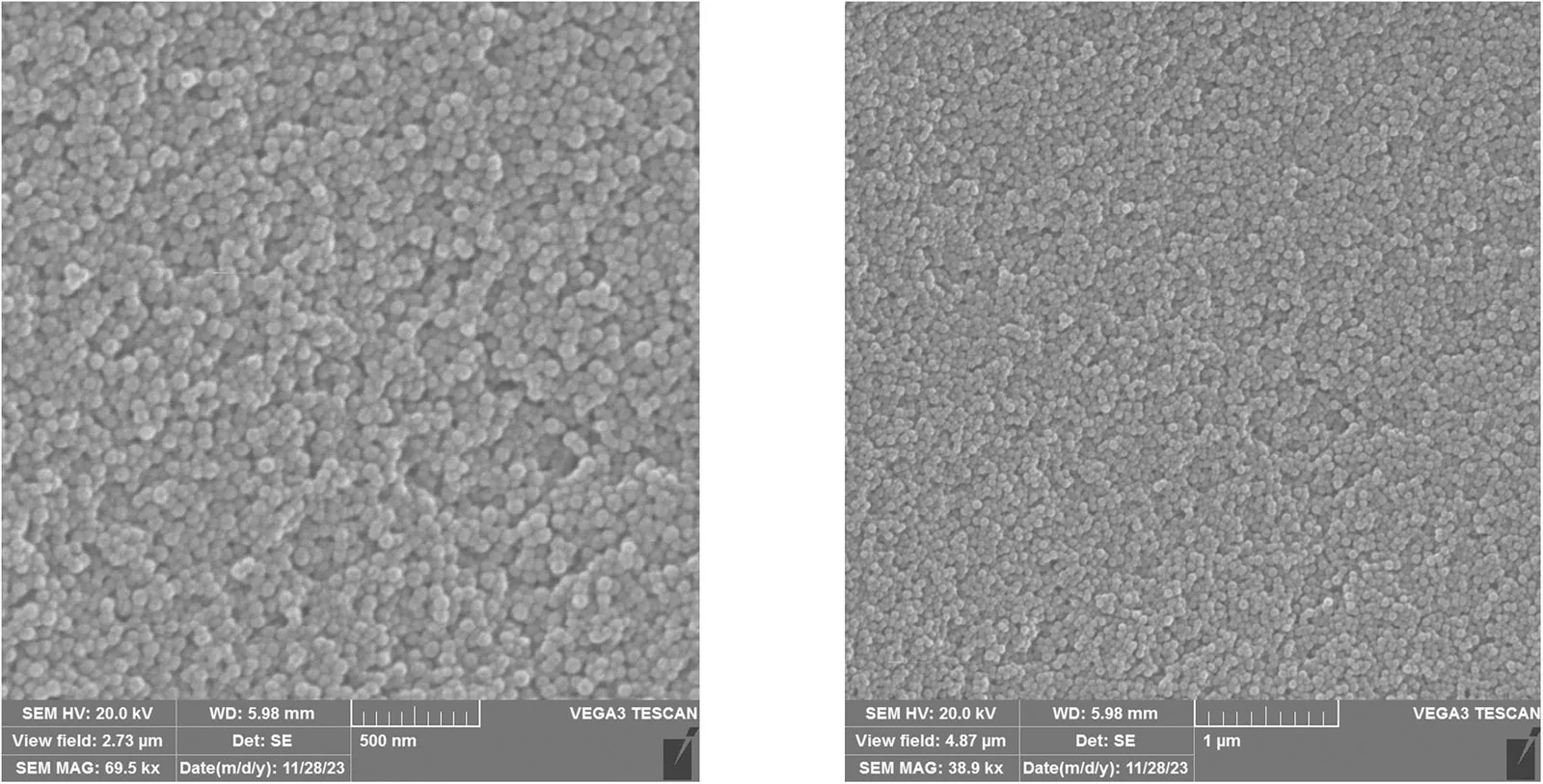
SEM image of PSNPs at different scales: 500 nm (left) and 1 μm (right). This image was prepared by the authors from the results of the present study.

**Fig 2:**
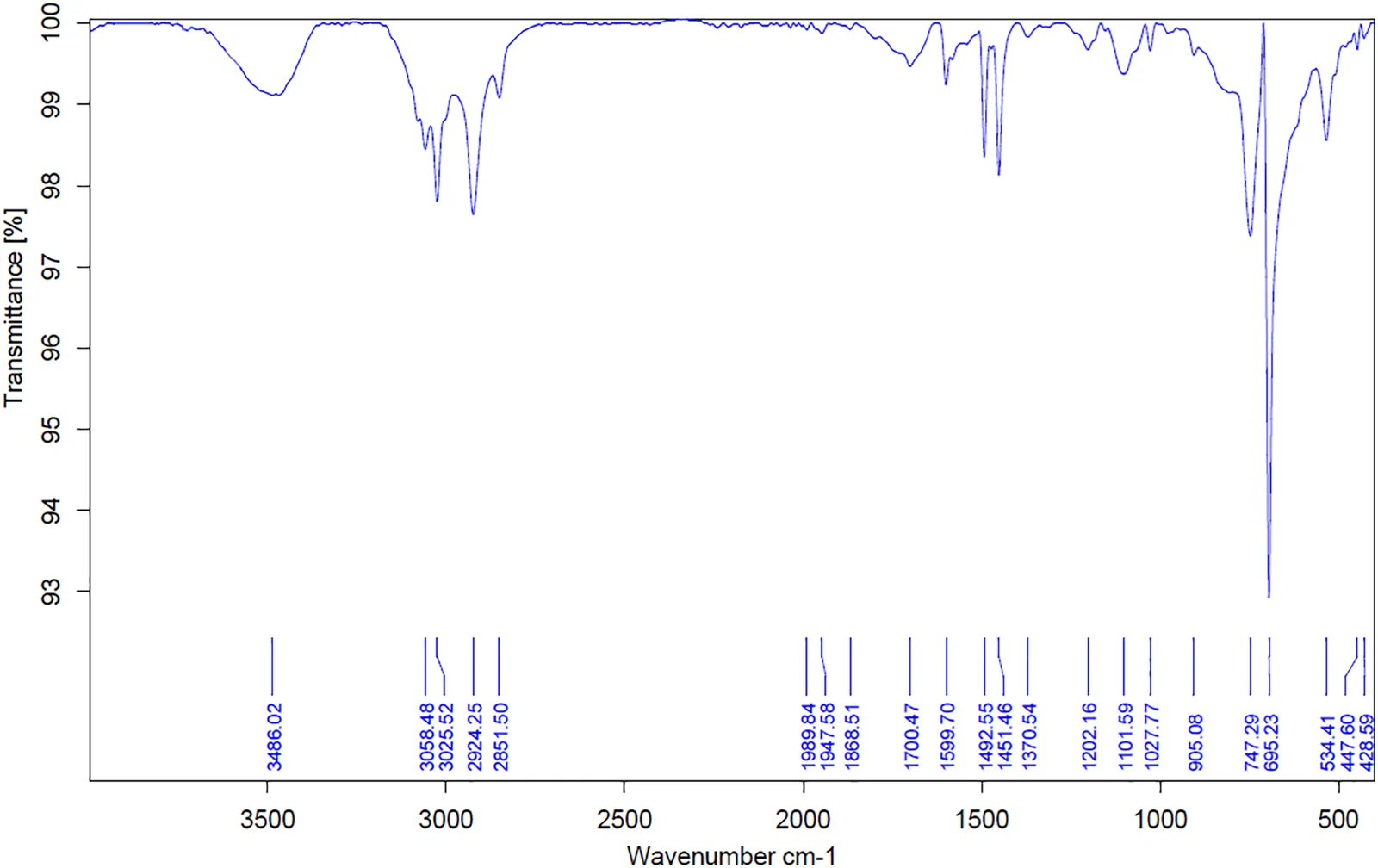
Characteristic FTIR peaks observed for PSNPs.

### 2.2. Plant cultivation and hydroponic setup

To circumvent potential interference from soil components and ensure rigorous control over environmental variables and nutrient uptake, a hydroponic experiment was conducted using specialized containers, each housing three individual pots. This specific configuration was designed to facilitate systematic sampling across three growth stages, ensuring that each pot provided a discrete specimen for temporal analysis (Fig 3). These containers measured 16 cm in both height and diameter, with a total volume capacity of 3 L. After transferring the spinach seedlings into the pots, each container was filled with Hoagland nutrient solution (as described by [40]). The nutrient solution was fully renewed once per week, while evaporative and transpirational water losses were compensated every two days by replenishing with deionized water. To maintain sufficient root oxygenation, air pumps connected via tubing continuously supplied oxygen to all containers.

**Fig 3:**
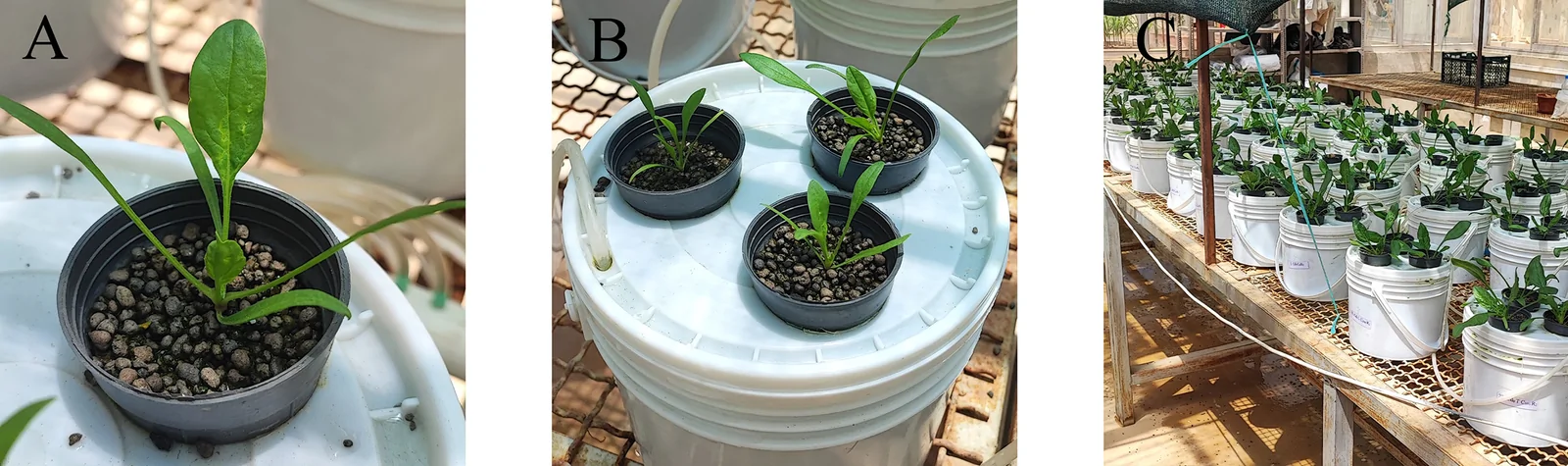
Experimental setup for spinach cultivation: (A) spinach seedlings grown in individual pots containing LECA; (B) arrangement of three spinach seedlings in containers with Hoagland nutrient solution; (C) overview of the greenhouse hydroponic cultivation system.

Spinach seeds (*S. oleracea,* Hachure variety) were surface-sterilized by soaking in a 2% sodium hypochlorite solution for 15 minutes, followed by three rinses with distilled water to eliminate any residual disinfectant. The sterilized seeds were then sown into seedling trays filled with perlite and placed in a controlled greenhouse environment (25°C and 60% relative humidity). Seedlings were irrigated daily, and after a 15-day growth period, they were transplanted into small pots containing LECA as the root substrate.

### 2.3. Experimental setup and treatment application

The experiment was carried out using a completely randomized factorial design with three independent biological replications (each pot serving as an independent experimental unit). The main experimental variables included: concentration of PSNPs, Cd levels, and duration of exposure. Six distinct PSNP concentrations (0, 10, 50, 100, 200, and 400 mg L^-1^), all with an average particle size of 62.3 ± 0.5 nm, were prepared and designated as NP_0_, NP_10_, NP_50_, NP_100_, NP_200_, and NP_400_, respectively. Foliar application of these solutions was carried out on a weekly basis throughout the growth period. The volume of spraying solution was adjusted according to plant growth stage, with approximately 40 mL applied per pot during the early growth stages when plants were smaller, and gradually increased to about 80 mL per pot in later stages as plant size increased. Accordingly, throughout each foliar application, mature plants receiving 80 mL per pot of the suspension were exposed to 0, 0.8, 4, 8, 16, and 32 mg of PSNPs at concentrations of NP_0_, NP_10_, NP_50_, NP_100_, NP_200_, and NP_400,_ respectively. To maintain uniform experimental conditions, plants in the control treatment (NP_0_) were sprayed with the same volume of distilled water. Before each application, the suspensions were sonicated in a bath-type ultrasonic cleaner at 50 W for 10 min to ensure proper dispersion and homogeneity. During spraying, the surface of each pot was covered with plastic film to prevent substrate contamination. The solutions were then applied until all aerial plant parts were uniformly wetted, ensuring even distribution of NPs across the entire shoot canopy.

Cd was applied at two levels: a control treatment without Cd addition (Cd_0_) and a Cd treatment (Cd_1_). In the Cd_1_ treatment, Cd was supplied as anhydrous Cd nitrate [Cd(NO_3_)_2_] (Merck, Germany), and concentrations were expressed based on elemental Cd. The final Cd concentration in the nutrient solution was 5 mg Cd L^-1^. A stock solution was prepared by dissolving 15.77 g of anhydrous Cd(NO_3_)_2_ in 500 mL of distilled water. Subsequently, 1 mL of this stock solution was added to each 3-L hydroponic container, resulting in the desired Cd concentration of 5 mg Cd L^-1^.

Three exposure durations were employed to evaluate treatment effects over time: 2 weeks (t_1_), 4 weeks (t_2_), and 6 weeks (t_3_). Spinach seedlings aged 15 days were subjected to the treatments and harvested at the end of each respective time point (t1, t_2_, and t_3_).

### 2.4. Measurements of plant growth parameters

At each experimental time point (t_1_, t_2_, and t_3_), one pot was removed from the corresponding hydroponic unit. Plants were carefully separated into shoot and root components at the collar region. Following thorough rinsing with deionized distilled water and subsequent washing with an EDTA solution, fresh biomass was immediately recorded. Subsequently, the plant tissues were oven-dried at 65 °C to eliminate moisture content, and their dry weights were determined using an analytical balance (AND EK-610i) with a precision of 0.01 g.

### 2.5. Quantification of elemental concentrations

Dried plant samples were finely ground, and elemental extraction was conducted via wet digestion following the method outlined by Allen *et al.,* [41]. Approximately 0.5 g of powdered plant material was transferred into Kendall digestion tubes. A mixture of nitric acid and hydrochloric acid in a volumetric ratio of 1:3 was added to each sample and allowed to react at room temperature for 24 hours to initiate digestion. Subsequently, samples were heated in a paraffin bath maintained at 100°C. During this digestion phase, hydrogen peroxide (2 mL aliquots) was added every 30 minutes, accumulating to a total volume of 6 mL, until the solution became visibly clear, indicating completion of the digestion process.

Post-digestion, the samples were filtered using Whatman No. 42 filter paper to remove particulate matter, and the filtrate volume was brought to 25 mL with distilled water. This prepared solution was employed for Cd analysis in both shoot and root tissues. To ensure analytical accuracy, reagent-grade chemicals and distilled water were used throughout. Calibration included blank samples prepared with distilled water and standard Cd solutions with concentrations ranging from 0.1 to 3 mg L^-1^. Metal quantification was performed using a Shimadzu AA-670 atomic absorption spectrophotometer, following the procedure described by Isaac and Kerber [42].

### 2.6. Calculation of Transfer Factor (TF)

The transfer factor (TF), also referred to as the mobility coefficient, quantifies the efficiency of metal translocation from the root system to the aerial parts of the plant, including shoots and leaves [43,44]. TF values were computed based on the following equation (Eq. 1):


$$\begin{array}{c} TF=\frac{concentrationinshoot}{concentrationinroot} \end{array}$$ (1)

### 2.7. Inclusivity in global research

Additional information regarding the ethical, cultural, and scientific considerations specific to inclusivity in global research is included in the Supporting Information (S2 Checklist).

### 2.8. Statistical analysis

The experiment was arranged as a factorial trial based on a completely randomized design with three replications. Statistical analyses were performed using SAS software (version 9.4), followed by mean comparisons using Tukey’s HSD test at P < 0.05. Results are expressed as mean ± standard error (SE). Microsoft Excel was used for data organization and graphical presentation, whereas Adobe Photoshop CS6 was employed for final figure preparation.

## 3. Results

### 3.1. Characteristics of PSNPs

Scanning electron microscopy (SEM) images clearly demonstrate the morphology of the synthesized PSNPs at high resolution. Analysis of these micrographs shows that the particles are predominantly spherical with a relatively smooth and uniform surface (Fig 1).

To investigate the hydrodynamic diameter and uniformity of the PSNP size distribution, dynamic light scattering (DLS) analysis was performed in three independent replicates. The Z-average hydrodynamic diameter was measured at 62.3 ± 0.5 nm.

Furthermore, the polydispersity index (PDI) was 0.118 ± 0.032, reflecting a narrow particle size distribution. Given that PDI values below 0.3 are typically associated with monodisperse systems, the results confirm a high degree of size uniformity among the three replicates.

The zeta potential of the PSNPs was −44.0 ± 0.46 mV (n = 3), indicating a strongly negative surface charge. The high absolute zeta potential value suggests good colloidal stability of the nanoparticle suspension and a low tendency toward aggregation under the measurement conditions.

The FTIR results for the PSNPs show that the absorption pattern of the sample matches the characteristic structure of polystyrene (Fig 2). The bands at 3058 and 3025 cm^-1^ are attributed to aromatic C–H stretching vibrations, while the bands at 2924 and 2851 cm^-1^ correspond to aliphatic C–H stretching in the polymer chain. The bands at 1599 and 1492 cm^-1^ are assigned to the C = C aromatic ring stretching vibrations. Furthermore, the presence of prominent peaks at 747 and 695 cm^-1^, which are characteristic of polystyrene, confirms the existence of monosubstituted benzene rings. Collectively, the FTIR spectrum is consistent with the characteristic chemical structure of polystyrene [45].

### 3.2. Spinach biomass under different foliar PSNP concentrations

This study aimed to assess the effects of varying concentrations of foliar-applied NPs on the dry weight of plant shoots and roots across three-time intervals (t_1_, t_2_, and t_3_). The corresponding results are illustrated in Fig 4. At time point t_1_, changes in the dry weight of both shoots and roots followed relatively similar trends across NPs concentrations. However, root biomass data exhibited greater variability compared to that of the shoots. At t_1_, both shoot and root dry weights showed their highest numerical values under NP_50_; however, differences among NP treatments were not statistically significant (p > 0.05).

**Fig 4:**
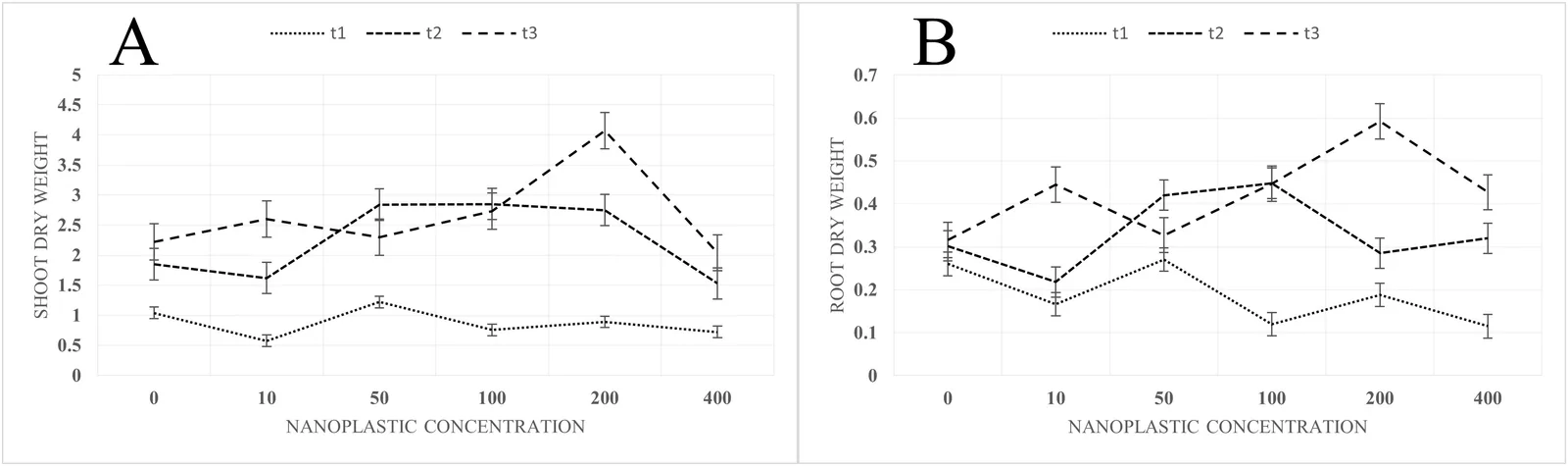
Dry weight of spinach shoot (A) and root (B) as a function of varying foliar PSNPs concentrations across different time point.

At time point t_2_, shoot and root dry weights showed similar response patterns up to the NP_50_ concentration, after which their trends diverged. The highest biomass increase was again observed at NP_50_, where shoot and root dry weights were 53.51% and 39.07% higher than the control, respectively. However, the increase was not statistically significant for either shoot or root dry weight (p > 0.05). As NP concentrations increased beyond NP_50_, shoot dry weight declined, whereas root dry weight showed a slight increase at NP_100_, followed by a decrease at NP_200_ to a value approximately 36.38% lower than that recorded at NP_100_. At NP_400_, the dry weights of both shoots and roots returned to levels statistically comparable to those of the control (*p* > 0.05).

In the final stage, the exposure duration was extended to six weeks (t_3_). During this period, the overall trends in shoot and root dry weights remained consistent. Unlike the previous time points, the highest biomass accumulation was observed at the NP_200_ concentration, where shoot and root dry weights increased by 83.33% and 87.34%, respectively, compared to the control (p < 0.05). However, further increasing the concentration to NP_400_ resulted in a reduction in shoot and root dry weights by 49.87% and 27.87%, respectively, relative to the NP_200_ treatment (p < 0.05).

### 3.3. Spinach biomass under different Cd levels

Simultaneously with NP application, a subset of samples was also exposed to Cd. Changes in shoot and root dry weights under these treatments were evaluated relative to the control (Fig 5). The results clearly indicate that Cd exposure had a markedly negative impact on plant biomass, leading to a significant reduction in both shoot and root dry weights (p < 0.05).

**Fig 5:**
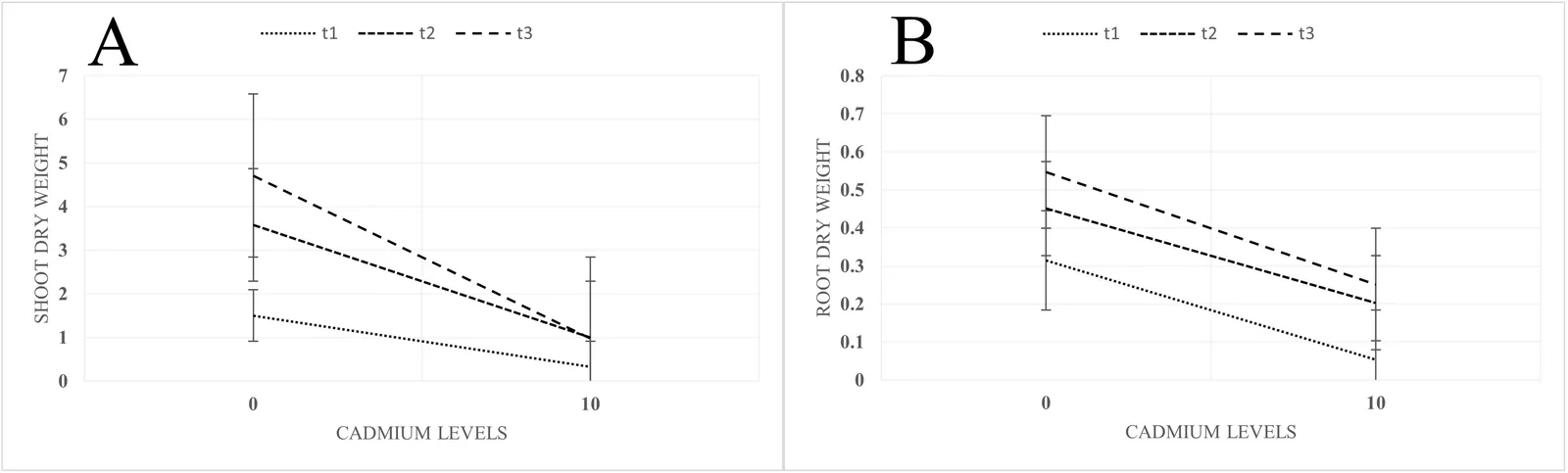
Dry weight of spinach shoot (A) and root (B) as a function of Cd levels across different time point.

Upon further and time-dependent examination, in the Cd_0_ treatment, shoot dry weight increased significantly over time; specifically, at t_2_ and t_3_, it increased by 138.66% and 214%, respectively, compared to t_1_ (p < 0.05). In contrast, under Cd_1_ treatment, shoot growth was markedly inhibited, with dry weight remaining nearly unchanged between t_2_ and t_3_ (p > 0.05), indicating growth suppression in response to Cd-induced stress.

Root dry weight exhibited a similar upward trend in the Cd_0_ treatment, with increases of 43.63% at t_2_ and 74.20% at t_3_ relative to t_1_, where the increase at t3 was statistically significant (P < 0.05), whereas the difference between t_2_ and t_1_ was not statistically significant (p > 0.05). In contrast, in the Cd_1_ treatment, the temporal increase in root dry weight was minimal and not statistically significant across the sampling intervals (p > 0.05).

### 3.4. Biomass response of spinach to PSNP and Cd co-exposure

In this section, the combined effects of NPs and Cd on the dry weight of plant shoots and roots were evaluated. The results are illustrated in Fig 6. As shown in the graphs, the trends in biomass changes for plant organs under similar Cd conditions (presence or absence) followed a relatively consistent pattern, although the absolute dry weight values differed between shoots and roots.

**Fig 6:**
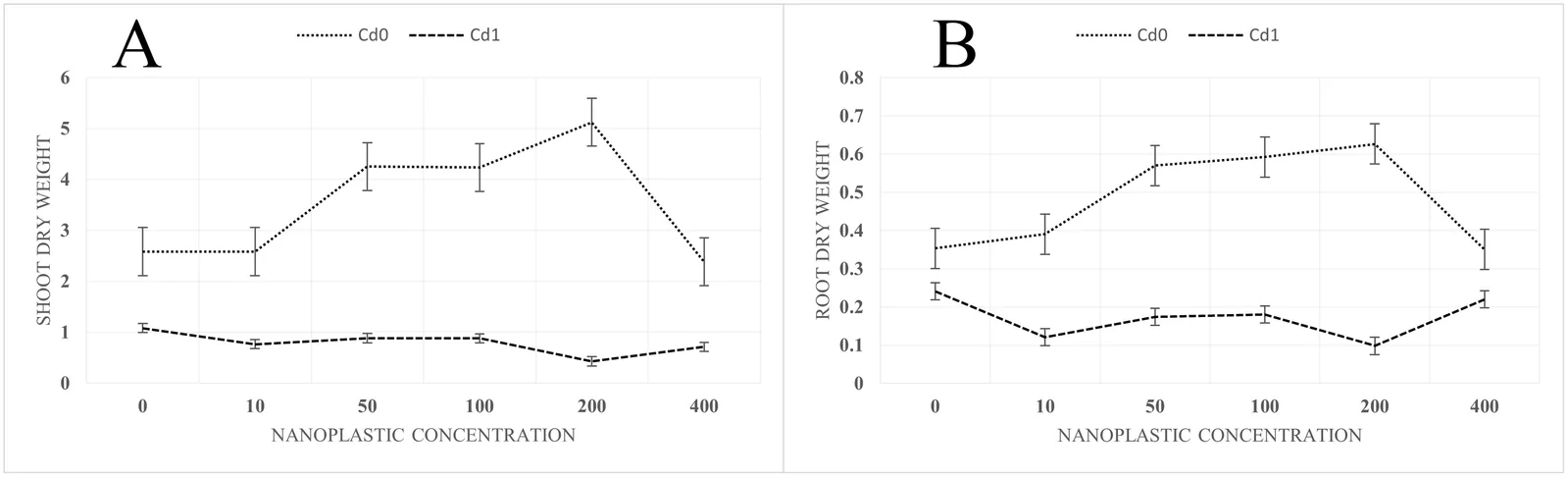
Dry weight of spinach shoot (A) and root (B) as a function of varying foliar PSNP concentrations under different Cd levels.

In the Cd_0_ treatments, increasing the NPs concentration up to NP_200_ resulted in maximum biomass accumulation. At this concentration, shoot and root dry weights increased by 98.44% and 77.33%, respectively, compared to the control (p < 0.05). However, at the highest concentration (NP_400_), plant growth declined significantly; shoot and root dry weights decreased by 53.51% and 44.08%, respectively, relative to NP_200_, approaching control levels (p < 0.05).

In contrast, in Cd_1_ treatments, plant growth was substantially inhibited (p < 0.05). Interestingly, at high NP concentrations (NP_400_), Cd toxicity appeared to be partially alleviated. At this level, shoot and root dry weights showed an increasing trend of 65.88% and 124%, respectively, compared to the NP_200_ treatment; however, these differences were not statistically significant (p > 0.05).

### 3.5. Effect of foliar PSNP concentrations on Cd accumulation in spinach

In another section of this study, the influence of varying NP concentrations on Cd concentration in plant tissues was assessed. The Cd concentrations in both shoots and roots are depicted in Fig 7. As shown in the graph, the presence of NP—even at low concentrations—led to an increase in Cd concentration in the shoot tissues. At the initial sampling time (t_1_), increasing NP concentrations had no statistically significant effect on Cd content in shoot (p > 0.05). However, at subsequent time points (t_2_ and t_3_), a marked increase in shoot Cd concentration was observed, peaking at NP_50_. At this concentration, Cd accumulation increased by 58.93% at t_2_ (p > 0.05) and 72.28% at t_3_ (p < 0.05) compared to the control. Interestingly, further elevation of NP concentrations beyond NP_50_ resulted in a decline in Cd content in the shoots, although the values remained higher than those of the control (p > 0.05).

**Fig 7:**
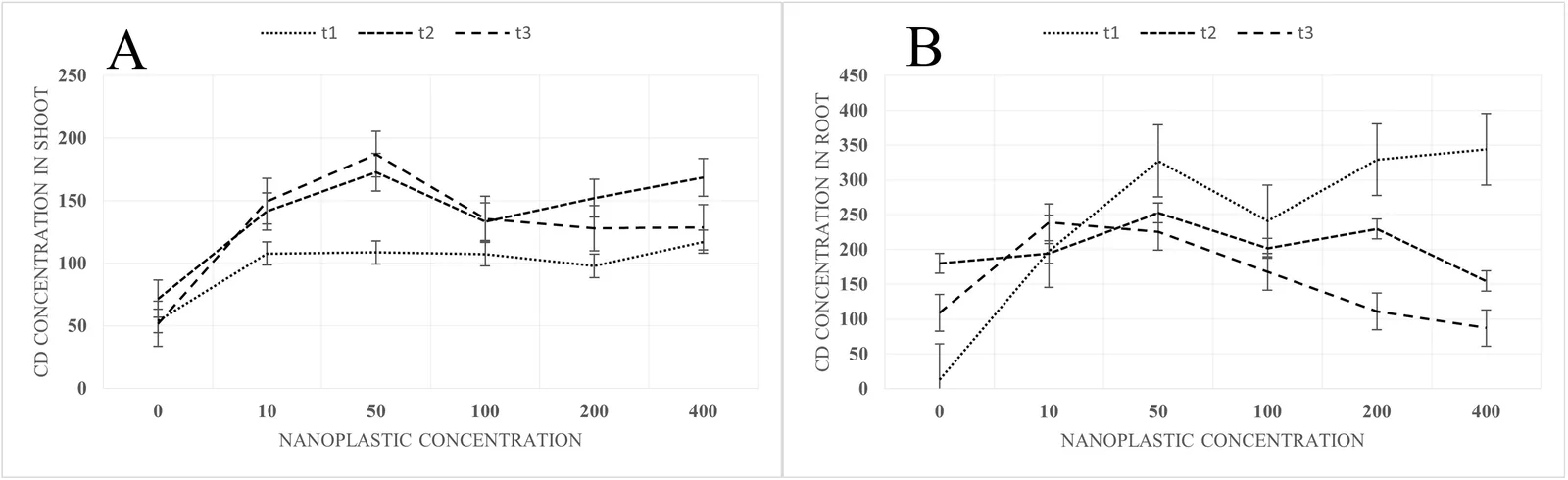
Cd concentration in spinach shoots (A) and roots (B) as a function of varying foliar PSNP concentrations across different time point.

Compared to the shoots, the pattern of Cd accumulation in roots displayed a distinct trend. The most pronounced impact of NP on root Cd levels was observed at the initial time point (t_1_), where increasing NP concentrations led to a significant rise in Cd accumulation (p < 0.05).

By t_2_, this pattern plateaued, showing only slight fluctuations in Cd levels despite further increases in NP concentration (p > 0.05). At the final time point (t_3_), a sharp increase in root Cd concentration was observed at NP_10_, with levels rising by approximately 119% relative to the control (p < 0.05). However, as NP concentrations continued to increase, root Cd levels gradually declined in an almost linear manner. At the highest concentration (NP_400_), Cd accumulation in roots decreased by approximately 63.03% compared to NP_10_ (p < 0.05).

### 3.6. Effect of exposure period on Cd content in spinach

Fig 8 depicts the effect of exposure duration on Cd concentration in different plant organs. As illustrated, Cd concentration in the shoots shows a slight initial increase, followed by stabilization over time (p > 0.05). In contrast, Cd levels in the roots decline progressively and in an approximately linear manner throughout the exposure period (p < 0.05).

**Fig 8:**
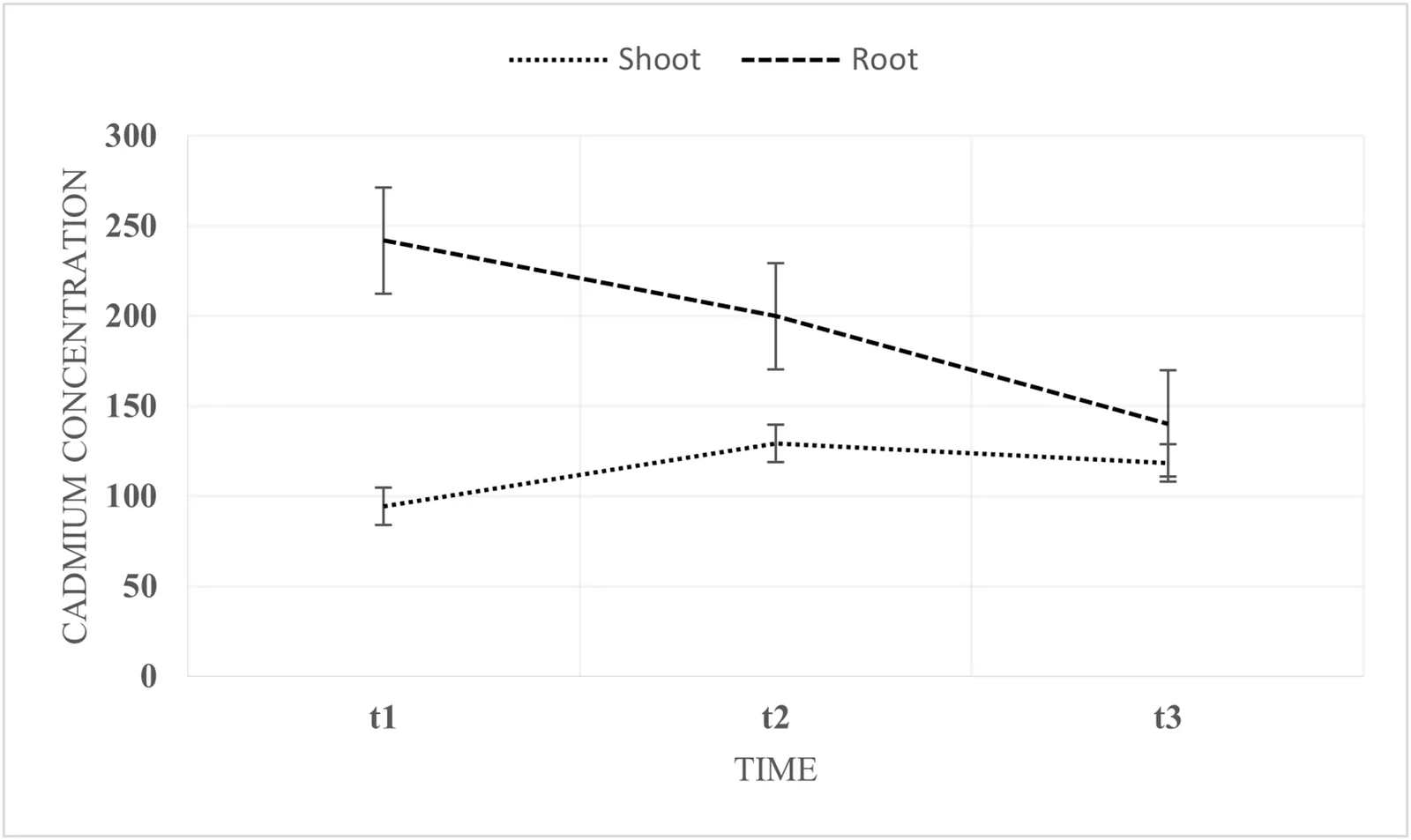
Cd concentration in spinach shoots and roots as a function of exposure duration.

### 3.7. Effects of foliar PSNP concentrations and exposure duration on Cd Transfer Factors (TF)

The Cd transfer factor (TF), defined as the ratio of Cd concentration in the shoot to that in the root, was affected by varying NP concentrations. Fig 9 illustrates changes in TF across three exposure durations and multiple NP levels.

**Fig 9:**
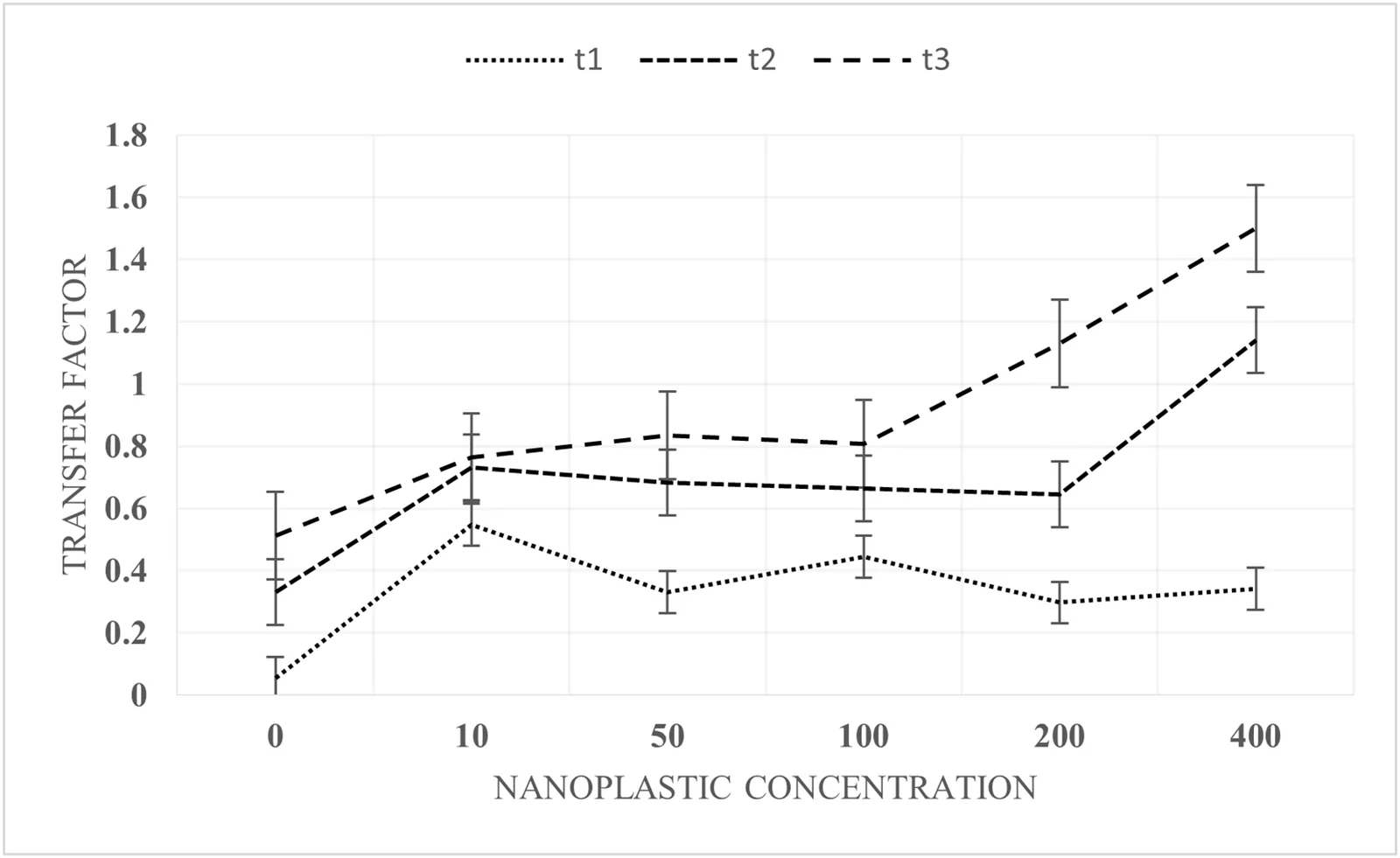
Cd transfer factor (TF) in response to varying foliar PSNP concentrations across different time point.

As shown, the presence of NPs—even at low concentrations—initially increased the TF, suggesting enhanced translocation of Cd from roots to shoots. At intermediate concentrations, TF values remained relatively constant (p > 0.05). However, with extended exposure durations and higher NP concentrations, TF values increased significantly, particularly at t_3_. At this time point, a marked elevation in TF was observed beginning at NP_100_ and above (p < 0.05).

The effect of exposure duration on the Cd transfer factor (TF) is illustrated in Fig 10. As depicted in the graph, TF values increased in an approximately linear manner over time. Specifically, at time points t_2_ and t_3_, TF values were elevated by 88.63% and 145.73%, respectively, compared to t_1_ (p < 0.05). This consistent upward trend suggests that Cd translocation from roots to shoots is progressively enhanced with prolonged exposure duration.

**Fig 10:**
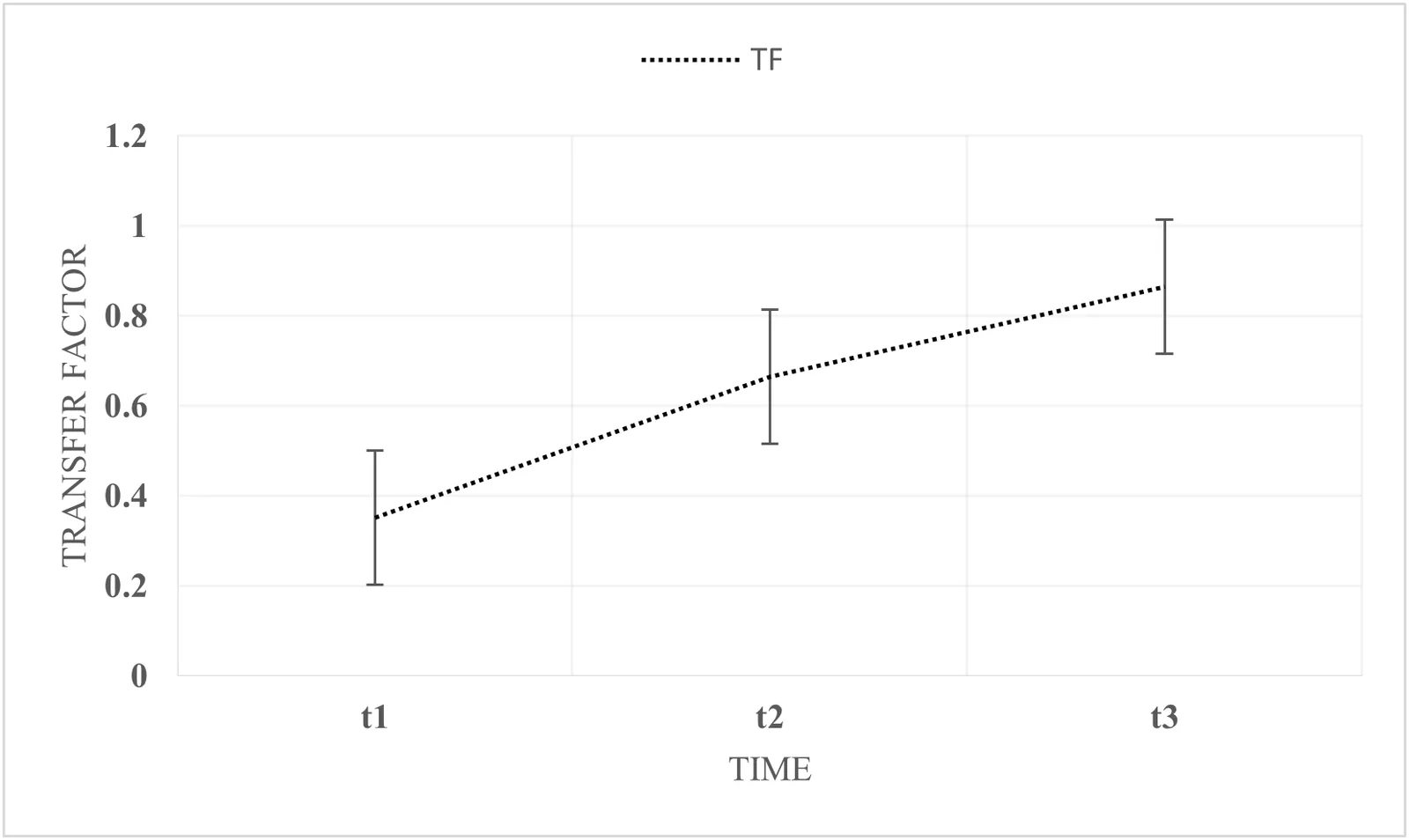
Cd translocation factor (TF) in spinach as a function exposure duration (t_1_, t_2_, t_3_).

## 4. Discussion

The graphical data revealed that the patterns of dry weight variation in both shoots and roots remained generally similar across various PSNP concentrations at all time points. The greatest increases in dry weight were observed at lower PSNP concentrations. In contrast, further increasing the concentration to NP_400_ resulted in a significant decline in dry weight, with values approaching those of the control. These findings are consistent with results reported in previous studies. Numerous investigations have demonstrated that NPs exert considerable effects on plant growth and physiology [46,47], with factors such as size, type, and concentration of the particles playing pivotal roles [18–20]. Among these, concentration appears to be one of the most critical parameters, as higher levels of NPs have been shown to cause adverse effects on plants. Several studies have reported significant reductions in both above- and below-ground biomass under high NP exposure [15,48]. For instance, in *Arabidopsis thaliana*, exposure to PS-SO_3_H and PS-NH_2_ nanoparticles led to a 50% decrease in fresh weight, accompanied by a 15% and 30% reduction in shoot and root length, respectively—effects that were predominantly observed at higher concentrations [15]. Similarly, polystyrene particles of 5 µm in size reduced both fresh and dry weights of bean plants at all tested concentrations, while 100 nm particles only affected biomass at 100 mg/L, showing no toxicity at 10 or 50 mg/L [49]. Exposure to 50 nm PSNPs at a concentration of 1 g/L for 72 hours led to a 41% reduction in root growth [16]. In another study, *Duckweed* exposed to amorphous polyethylene particles at concentrations ranging from 10 to 100 mg over seven days experienced a reduction in root length [46,50]. As observed in the current experiment and corroborated by prior research, high concentrations of NPs inhibit plant growth. This growth inhibition at elevated concentrations may be attributed to the generation of reactive oxygen species (ROS), interference with nutrient uptake, and cellular damage [37,51]. On the other hand, lower concentrations may exert stimulatory effects on plant growth. For example, the addition of MPs to soil was found to enhance tomato plant growth, although it was also associated with delayed fruit development and reduced yield [52]. Taken together, the findings of this study indicate that the effects of PSNP on spinach growth are concentration-dependent: lower concentrations promote growth and increase dry biomass, whereas higher concentrations exert inhibitory effects. These results are consistent with previous studies and emphasize the critical role of concentration in determining the overall impact of NPs on plant systems.

Upon exposure to Cd, a significant reduction in biomass was observed compared to the control. Further analysis revealed that in the Cd_0_ treatment, dry weight significantly increased over time. In contrast, in the Cd_1_ treatment, plant growth was markedly suppressed, and time had no significant effect on biomass accumulation. Previous studies have consistently demonstrated that increasing Cd concentrations in soil leads to significant reductions in both fresh and dry weights of spinach [53,54]. This biomass decline is often accompanied by morphological alterations in root architecture, including shortening of the primary root and modifications in root branching patterns. These structural changes restrict the plant’s ability to efficiently absorb water and nutrients, ultimately exacerbating stress responses [53–55]. Other contributing factors to growth inhibition under Cd stress include impaired water and nutrient uptake, reduced photosynthetic activity, and inhibition of cell division [56]. Cd toxicity is commonly associated with leaf chlorosis, decreased chlorophyll synthesis, and lower photosynthetic efficiency [57,58]. The findings of this study suggest that, unlike PSNP—which may promote plant growth at lower concentrations—Cd exhibits deleterious and growth-suppressive effects even at low levels. This contrast highlights the inherently more toxic and inhibitory nature of Cd compared to PSNP.

This study also examined the interactive effects of Cd and PSNPs on spinach growth. The results indicated that Cd exhibited a dominant inhibitory effect compared to PSNP. In plants exposed to Cd, changes in shoot and root dry weights were negligible across different concentrations of PSNPs. However, at the highest concentration (NP_400_), a slight improvement in biomass, particularly root dry weight, was observed. In contrast, plants not exposed to Cd showed noticeable changes in biomass across PSNP concentrations, with both shoot and root dry weights increasing up to NP_200_, followed by a sharp decline at NP_400_. A study by Pinto-Poblete *et al.,* [26] demonstrated that the simultaneous presence of MPs and Cd significantly inhibited strawberry plant growth, leading to reduced stem diameter, overall plant development, and root elongation. However, conflicting results were reported by Zong *et al.,* [59] in a hydroponic experiment with wheat seedlings. Their findings suggested that polystyrene MPs, when combined with HMs such as Cd and copper, increased chlorophyll content and mitigated oxidative stress by limiting the accumulation of reactive oxygen species (ROS). These observations suggest that at certain concentrations, PS may attenuate the apparent phytotoxic effects of Cd. However, whether this response is associated with changes in Cd bioavailability requires further direct investigation. Similarly, another study showed that although both Cd and PSNPs-Cd reduced plant growth, the adverse effects were more severe under Cd treatment alone. Specifically, Cd alone reduced dry biomass by 31.3%, whereas the combined Cd–PSNPs treatment resulted in only a 14.9% decrease [37]. These findings imply that PSNPs may mitigate Cd toxicity under specific exposure conditions; however, the present data do not directly demonstrate whether this effect results from PSNP–Cd interactions or changes in Cd availability in the growth medium. A comparable trend was observed in microalgae, where co-exposure to copper and MPs reduced specific growth rate; however, the reduction was less pronounced and not statistically significant compared to Cu alone [60]. Consistent with these observations, previous studies have shown that the presence of PSNPs under Cd stress, compared with Cd alone, can lead to moderate improvements in Pn, Gs, Tr, chlorophyll content, and nitrogen uptake in wheat [37]. Overall, the present findings suggest that under combined exposure to PSNP and Cd, the growth-inhibiting effect of Cd remains dominant. PSNPs appeared to partially alleviate Cd-induced adverse effects under specific conditions, particularly at higher concentrations. PSNPs, the extent and nature of these interactions depend on plant species, environmental factors, and the physicochemical properties of the particles involved.

In another part of this study, the effect of different PSNPs concentrations on Cd accumulation in the plant was evaluated. The observations revealed that low concentrations of PSNPs led to an increase in Cd uptake by the plant. However, as PSNPs concentrations continued to rise, Cd concentrations gradually declined or stabilized. Similar patterns have been reported in other studies. For instance, lower levels of Cd in solutions containing PSNPs-Cd have been associated with increased Cd bioaccumulation in wheat seedlings, suggesting that PSNPs may act as carriers facilitating Cd transport into plant tissues [37]. MPs have been recognized as potential vectors for HMs, capable of transporting metals to the rhizosphere and enhancing their bioavailability and root uptake [2,34]. Related studies have demonstrated that metals such as lead, Cd, and zinc can adsorb onto the surface of polyethylene terephthalate (PET) MPs in the wheat rhizosphere. This adsorption increases the metals’ residence time in the root zone, thereby promoting their bioaccumulation in plant tissues [36]. However, some contradictory findings have also been reported. For example, Tang *et al.,* [61] observed that polyethylene MPs reduced Cd uptake in rice roots. Overall, the results show that low concentrations of PSNPs were associated with increased Cd accumulation in plant tissues, whereas this effect was less evident at higher PSNP concentrations. Although this pattern may be consistent with a potential carrier-like role of PSNPs, direct evidence for NP-mediated Cd transport was not obtained in the present study. This complex behavior highlights that the impact of PSNP on HM uptake is highly dependent on factors such as concentration, plastic type, plant species, and environmental conditions.

In the subsequent phase of this study, the effects of varying PSNPs concentrations and exposure durations on the Cd transfer factor (TF) were evaluated. The results indicated that even at low PSNP concentrations, an initial increase in Cd translocation from roots to shoots occurred. As both concentration and exposure duration increased, TF values continued to rise steadily. This near-linear trend suggests that prolonged exposure enhances Cd movement to shoot plant. Such a pattern is consistent with findings from previous studies, some of which have shown that plants tend to accumulate HMs in their shoots over time. For instance, a similar study reported that arsenic translocation to rapeseed stems was influenced by exposure to PMMAMP particles [2]. However, contradictory findings have also been reported. Some studies have shown that MPs do not significantly affect the translocation of HMs to aerial tissues. For example, research has demonstrated that combining polylactic acid (PLA) with Cd reduced toxicity to the plant, whereas combining polyethylene (PE) with Cd exacerbated it [62]. Moreover, another study found that polyethylene MPs reduced Cd uptake in rice roots [61]. The results of the present study showed that the Cd TF increased with both PSNP exposure and time, particularly at higher concentrations and after extended exposure periods. This trend indicates that PSNP exposure was associated with increased Cd accumulation in above-ground plant parts, potentially raising concerns regarding metal accumulation in edible tissues. However, the magnitude and nature of this effect appear to depend on the type of plastic, duration of exposure, and plant species involved.

## 5. Limitations of this study

Despite the significant findings of this study, the use of a hydroponic system should be considered when interpreting the results. Although this system provided controlled exposure conditions for evaluating PSNP–Cd interactions in spinach, it cannot fully simulate the complexity of soil environments. Soil-related factors, such as organic matter, microbial activity, pH, and sorption processes, may alter the behavior, mobility, and bioavailability of PSNPs and Cd, thereby influencing plant uptake under realistic agricultural conditions.

Another limitation is that foliar PSNP uptake was not directly assessed. Therefore, the present results cannot clearly distinguish between PSNP retention on the leaf surface and actual internalization into leaf tissues. Future studies using standardized washing procedures, tracer-based labeling, or imaging approaches would help better characterize foliar PSNP uptake and clarify its possible interaction with Cd transport.

Based on these considerations, future research should focus on soil-based or soil–plant systems, include a broader range of PSNP concentrations and particle sizes, and integrate comprehensive biochemical, physiological, and molecular assays. Such approaches would provide deeper insight into the mechanisms underlying plant responses to combined PSNP and Cd exposure and help clarify their effects on Cd uptake and root-to-shoot translocation.

## 6. Conclusions

This study demonstrated that the concentration of PSNPs, Cd, and the duration of exposure significantly influenced the growth and chemical composition of spinach plants.

After six weeks of exposure (t_3_), the dry biomass of shoots increased up to a PSNP concentration of NP_200_, but sharply declined at higher concentrations. However, in treatments exposed to Cd, plants experienced severe growth inhibition, indicating a dominant phytotoxic effect of Cd. Under these conditions, different PSNP concentrations had no significant impact on plant growth, suggesting that the toxicity of Cd overrode any potential effects of the NPs. Notably, at the highest PSNP concentration (NP_400_), a modest recovery in dry biomass was observed.

Temporal analysis of exposure effects revealed that after two weeks (t_1_), plants exhibited only mild responses to PSNP and Cd, with negligible effects on growth and biomass. The most pronounced impacts of pollutant exposure appeared after four and six weeks (t_2_, t_3_), indicating that prolonged exposure played a key role in shaping plant responses.

Overall, increasing PSNP concentrations—especially at lower levels—led to higher Cd accumulation in plant tissues, with the highest Cd concentration in shoots observed at NP_50_. In contrast, Cd accumulation in roots was more dependent on exposure duration, with prolonged exposure resulting in decreased Cd content, possibly due to increased translocation to shoots.

As PSNP concentration and exposure duration influenced Cd uptake, these variables also significantly affected the Cd transfer factor (TF). Both increasing PSNP levels and longer exposure times independently enhanced TF, suggesting that higher NP concentrations facilitate Cd transfer to edible plant parts. Additionally, older plants tended to accumulate more Cd in their shoot.

This study offers valuable insights into the interactive effects of PSNP concentration, Cd contamination, and exposure duration on plant physiology. The findings have important implications for environmental pollution management and sustainable agricultural practices, highlighting the complex interactions between NPs, toxic chemicals, and plant responses.

These findings raise serious concerns regarding the safety of agricultural products, as the increased accumulation of Cd in spinach shoots, mediated by PSNPs, suggests that the quality of edible plant tissues is significantly compromised by the interactive effects of emerging NPs/MPs, and HMs. Such uptake patterns threaten to escalate human dietary exposure to toxic metals, potentially pushing contaminant levels beyond established safety thresholds, even at relatively low concentrations of particulate pollutants. Furthermore, the results indicate that conventional frameworks for predicting phytotoxicity—which typically evaluate single contaminants in isolation—may underestimate the actual risks present in complex agroecosystems. By altering Cd bioavailability and accelerating its translocation from roots to aerial tissues, PSNPs shift the established paradigms of metal distribution within plants. Consequently, to safeguard both public health and agricultural sustainability, it is imperative that future risk-mitigation strategies transition toward a multi-contaminant perspective.

## Supporting information

S1 Data Data supporting this research. (XLSX)

S1 File Inclusivity in global research. (DOCX)
